# Psychological Effects of the COVID-19 Pandemic on Hungarian Adults

**DOI:** 10.3390/ijerph17249565

**Published:** 2020-12-21

**Authors:** Csanád Szabó, Judit Pukánszky, Lajos Kemény

**Affiliations:** 1 Department of Dermatology and Allergology, University of Szeged, 6720 Szeged, Hungary; kemeny.lajos@med.u-szeged.hu; 2 Institute of Psychology, University of Szeged, 6720 Szeged, Hungary; pukanszky.judit@gmail.com; 3 The Hungarian Centre of Excellence for Molecular Medicine University of Szeged—HCEMM-USZ Skin Research Group, Department of Dermatology and Allergology, University of Szeged, 6720 Szeged, Hungary; 4 The Hungarian Centre of Excellence for Molecular Medicine (HCEMM Ltd.), 6723 Szeged, Hungary

**Keywords:** COVID-19 pandemic, questionnaire design, psychological stress, coping skills, health status, depression, anxiety

## Abstract

We aimed to explore psychological effects of the coronavirus pandemic on Hungarian adults in the time of the national quarantine situation in May 2020.We conducted a cross-sectional observational study with the use of an anonymous online questionnaire that consisted of 65 items. The following measuring instruments were used: Perceived Stress Scale (PSS-10); The General Anxiety Disorder Assessment (GAD)-2; The Patient Health Questionnaire (PHQ)-2; European Quality of Life Visual Analogue Scale (EQ-VAS); Self-administered inventory of complaints (Hungarian questionnaire); Shortened (Hungarian) version of the Ways of Coping Questionnaire; 2 open-ended questions to examine the participants’ mood and ways of coping during the pandemic. The data of 431 participants were analyzed, their average age was 47.53 ± 11.66 years, and the percentage of females was 90%. The mean of participants’ scores were the following: 19.34 ± 7.97 for perceived stress, 73.05 ± 21.73 for health status, and 8.68 ± 4.65 for neurotic complaints. Thirty-four and one-tenth percent of participants were depressed, 36.2% were anxious, and they tended to use problem-focused coping strategies more frequently than emotion-focused ones. We found significant correlations between all of the seven examined psychological variables. Our results highlight the importance of stress management in the psychological support of healthy adults in quarantine situation caused by the coronavirus pandemic.

## 1. Introduction

Coronavirus disease 2019 (COVID-19) was declared a pandemic by the WHO on 11 March 2020 [1]. To manage the pandemic, the government of Hungary declared a quarantine situation in the country on 11 March [2]. The lockdown was eased on 18 May, but social distancing measures (one and a half meters was to be kept) continued to apply, and a mask still had to be worn when shopping and on public transport [3]. On 29 May, restaurants, cafes, bakeries, and buffets were allowed to serve food and drinks indoors [3].

The quarantine situation brought about psychological effects on different populations worldwide. It may have raised levels of perceived stress, anxiety, and depression in many countries [4,5,6,7,8,9,10,11,12,13].

Perceived stress can be defined as an understanding of a person of the amount of stress he or she is exposed to in a given point of time or in a specific period [14]. Bäuerle et al. [15] assessed mental health and health status of 15,037 German adults before and after the COVID-19 outbreak. The level of distress became higher after the outbreak compared to before the outbreak: the frequency of elevated levels of distress changed from 51.8% to 65.2% of respondents. Their results indicate an increased prevalence of mental health burden during the COVID-19 pandemic [15].

There are very brief questionnaires which are useful in screening for symptoms of depression and anxiety. The Patient Health Questionnaire (PHQ)-2 consists of two questions which inquire about the degree to which a person has experienced depressed mood and anhedonia over the past two weeks [16,17]. The aim of using the scale is to screen for depression, not to establish a psychiatric diagnosis or to examine the severity of depression [16,17]. The General Anxiety Disorder Assessment (GAD)-2 contains two questions about core symptoms of generalized anxiety disorder, feeling nervous, and a perceived incapacity to stop worrying or to control it [16,17].

The new challenges brought about by the pandemic posed certain challenges to populations globally. Coping skills became essential in addressing these new threats.

Forms of coping can be classified into two categories: problem-focused strategies usually concentrate efforts towards fixing a stressful problem, while emotion-focused strategies concentrate on minimizing the emotional outcomes of the problem using strategies, for example, as endurance, avoidance, or venting to someone else [18].

Duan et al. [18] examined the mental health status of adolescents and children in China during the outbreak, and they found that increased levels of anxiety correlated positively with emotion-focused coping style, and negatively with problem-focused coping style. The researchers highlighted that increased levels of depression was also associated similarly with the two categories of coping styles. Smartphone addiction and internet addiction both correlated positively with the frequency of depressive and anxiety symptoms [18].

Fu et al. [19] investigated psychological reactions to the pandemic of 1242 Wuhan residents. Regarding their study participants’ coping styles, approximately 70.2% of residents have actively responded to the pandemic situation by participating in activities, talking with others about worries, and looking on the bright side, and 29.8% of them relied on passive coping styles, such as escapism, smoking, and depending on others. The authors emphasize that physical exercise can divert attention from the pandemic situation and reduce panic; therefore, it is a positive coping mechanism in the face of the new challenges arisen from the pandemic [19].

Li [20] examined psychosocial responses to the pandemic of 1109 Chinese participants, and revealed that people using problem-focused coping were at higher risk of developing post-traumatic stress disorder (PTSD) than those who used emotion-focused coping; and participants using emotion-focused coping reported higher psychiatric morbidity than those who used both emotion and problem coping strategies. Li stated that it should be considered that the effect of the same strategy may vary at different period of the COVID-19 outbreak, and that both emotion-focused and problem-focused coping strategies were important and should be used at the same time to cope with potential negative mental health outcomes during the pandemic [20].

The level of the personality trait of neuroticism in individuals can also be an important factor in reacting to the threats posed by the pandemic.

People who score high on neuroticism: are more likely than the average person to experience such feelings as anxiety, anger, guilt, and depression; respond poorly to environmental stress; are likely to interpret ordinary situations as threatening, and can experience minor frustrations as hopelessly overwhelming; are often self-conscious and shy; and they may have trouble controlling urges and impulses when feeling upset [21]. Neuroticism has tremendous public health implications, impacting a multitude of psychopathological and physical health care concerns [22].

We aimed to explore certain psychological effects (on the levels of perceived stress, depression and anxiety, use of coping strategies, number of neurotic complaints, subjective health status, subjective opinions regarding their mood and coping skills) of the coronavirus pandemic on Hungarian adults in the time of the national quarantine situation in May 2020.

## 2. Materials and Methods

### 2.1. Course of the Project

We explored the psychological effects of the COVID-19 pandemic among Hungarian adults in COVID-19 lockdown between 7–20 May 2020 in the frame of a cross-sectional, observational exploratory study with the use of an anonymous online questionnaire that consisted of 65 items.

The course of the project began with making the questionnaire using Google Forms. The link of the online questionnaire was sent to three public groups via the social media website Facebook. By clicking on the sent link, all participants were able to read information about the project and about the consent to participate in it. Then the participant was able to give consent to participate in the project. After giving consent, the participant was able to start filling out the questionnaire. If the participant indicated that he/she did not want to give consent to the study, the website would have indicated that the participation of him/her has ended in the project. Hungarian people over the age of 18 were included in our study. For a participant, completing the online questionnaire took approximately 10–15 min. Anonymous answers were automatically saved. The participants were given code numbers based on the order in which they had filled out the questionnaire. The data was analyzed by the researchers.

We registered our study with the ClinicalTrials.gov Protocol Registration and Results System (Identifier: NCT04426266) [23].

All subjects gave their informed consent for inclusion before they participated in the study. The study was conducted in accordance with the Declaration of Helsinki, and the protocol was approved on 29 April 2020 by the Medical Research Council of Hungary (ETT), National Scientific and Ethical Committee (TUKEB)(Project identification code: IV/3484-2/2020/EKU).

### 2.2. Measuring Instruments

The first six items of the questionnaire recorded sociodemographic factors (age, sex, city of residence, marital status, level of education, employment status).

Perceived stress was measured with the Perceived Stress Scale (PSS-10) [24,25]. Description: 10 items about thoughts and feelings that characterize a person’s perception of stress based on the previous month. Items are answered on a 5-point scale with a range from 0 (never) to 4 (very often) and scores range from 0 to 40. Cronbach’s alpha value of this instrument was 0.934, which indicates a high level of internal consistency.

Level of anxiety was explored with The General Anxiety Disorder Assessment (GAD)-2 [17]. Description: 2 items to examine the level of anxiety based on the past 2 weeks. Items are answered on a 4-point scale with a range from 0 (not at all) to 3 (nearly every day). The points of the questionnaire range from 0–6. A score of 3 is the preferred cut-off point to identify possible cases for generalized anxiety disorder. Cronbach’s alpha value of this instrument was 0.879, and this indicated a good reliability score.

Level of depression was measured with The Patient Health Questionnaire (PHQ)-2 [17]. Description: 2 items to examine the level of depression based on the past 2 weeks. Items are answered on a 4-point scale with a range from 0 (not at all) to 3 (nearly every day). The points of the scale range from 0 to 6. If the score is 3 or greater, depressive disorder is likely for the respondent. Cronbach’s alpha value of this instrument was 0.852, which indicates a high level of internal consistency.

Self-reported health state was explored with the European Quality of Life Visual Analogue Scale (EQ-VAS) [26]. Description: 1 item to examine health status. Participants are asked to rate their health status on a vertical visual analogue scale, where points range from 0 (“the worst health you can imagine”) to 100 (“the best health you can imagine”).

Number of complaints were measured with the Self-administered inventory of complaints (Hungarian questionnaire) [27]. Description: 20 items to measure number of neurotic complaints. For each item of the questionnaire, 0 point or 1 point can be given to the respondent based on whether he/she had or did not have a specific complaint. Questions investigate neurotic complaints, such as emotional volatility, sleep quality, daytime sleepiness, vigilance, headache, vertigo, and gastrointestinal symptoms. If the score is 10 or greater, neurotic personality trait is likely for the respondent. Cronbach’s alpha value of this instrument was 0.836, which indicates a high level of internal consistency.

Strategies in coping with stressful situations were explored with the Shortened (Hungarian) version of the Ways of Coping Questionnaire [28,29]. Description: 22 items to measure cognitive or behavioral strategies in coping with stressful situations. The questionnaire contains seven subscales, two of them (problem analysis, goal-oriented behavior) measures problem-focused coping, and five of them (emotion-based behavior, adaptation, asking for help, seeking emotional balance, withdrawal) measures emotion-focused coping. Items are answered on a 4-point scale with a range from 0 (not used) to 3 (used a great deal). The points of the scale range from 0 to 21 on the problem-focused coping subscale, and 0–45 on the emotion-focused coping subscale. Percentage values for coping subscales were calculated as follows: the mean value of each of the seven subscales were divided by the maximum score for that specific subscale to obtain a number that shows the average percentage of scores for that given subscale. Cronbach’s alpha value of this instrument was 0.679.

Psychological effects of the coronavirus pandemic on the participants’ mood and ways of coping with difficulties arising from the pandemic were investigated with 2 open-ended questions (that were not obligatory to answer) in connection with psychological effects of the coronavirus pandemic (its effects on the participants’ mood and ways of coping with difficulties arising from the pandemic). Qualitative results regarding the two open-ended questions are not published in the present manuscript.

### 2.3. Statistical Analyses

Descriptive statistics and statistic tests were calculated with SPSS 17.0 software (IBM, Armonk, NY, USA). Internal consistency reliability of the scales was measured with Cronbach’s alpha. We tested seven variables for normality with the Kolmogorov–Smirnov test: levels of perceived stress, depression and anxiety, number of neurotic complaints, subjective health status, problem-focused coping, and emotion-focused coping. The analyzed variables were not normally distributed. Therefore, we used the Spearman rank-order correlation coefficient, a nonparametric measure to examine associations between the seven mentioned variables.

We intended to compare certain variables’ results of our study with several publications’ main outcomes where the authors used the same measuring instruments as we did, details of these studies are illustrated in Table 1. We did not have the datasets of the cited articles, only means of the variables that we wished to compare with our results, and because of this we could not use independent sample *t*-tests. However, one-sample *t*-test is suitable to compare a sample mean to a specific value [30,31,32]. We chose to use one-sample *t*-test to compare data of four variables (level of perceived stress, depression and anxiety, subjective health status) to other publications’ score of the same variable. The data we wanted to analyze with using a one-sample *t*-test needed to meet four assumptions to be eligible for analyzation with a one-sample *t*-test [33]. The first assumption was met, which was that the four dependent variables should be measured at the interval level. The second assumption was also met, which was that there was no relationship between the observations. The third assumption, that there should be no significant outliers, was met for three examined variables, but it was violated for the fourth variable: subjective health status. Therefore, we eliminated the significant outliers of this variable (data of 13 participants) to be able to meet the third assumption of the test for all four variables. The fourth assumption of the test was not met, which was that dependent variables should be approximately normally distributed. However, it is important to mention that the one-sample *t*-test is robust to violations of normality to a certain degree, which means that the assumption can be a little violated and still provide valid results [33]. Based on the available data, we decided to use one-sample *t*-test to compare our means among four variables to other studies’ means.

### 2.4. Participants

The online questionnaire of our study was completed by all participants (*n* = 441) in May 2020. 97% of participants (*n* = 428) completed it between 7 and 15 May, and 3% (*n* = 13) between 19 and 20 May. Data from 10 individuals were excluded from the analysis (nine of them did not consent to participate in the study by marking it at the beginning of the questionnaire, and one completed the questionnaire at age 17). Therefore, the data of 431 participants wasanalyzed.

Attributes of participants areshown in Table 2. They reported 151 locations as their places of residence in Hungary, the most frequent of whichwere the following: Budapest (38%), Győr (1.9%), Székesfehérvár (1.9%), Miskolc (1.6%), and Szeged (1.6%).

## 3. Results

**Figure 1:**
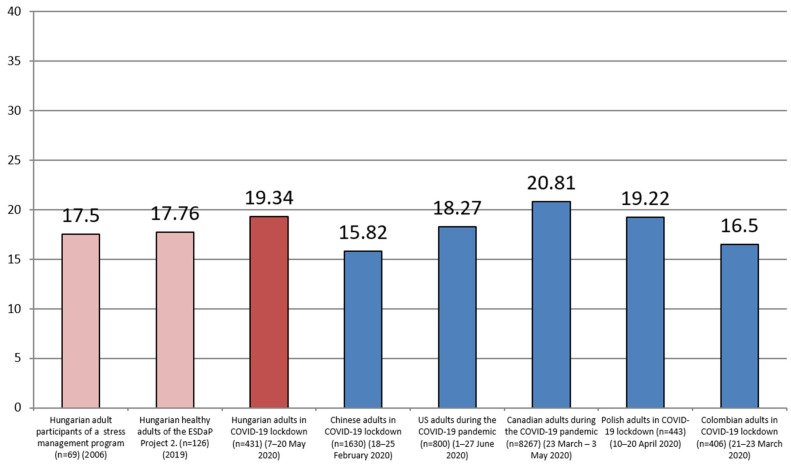
Perceived stress results (measured with the 10 item Perceived Stress Scale) of Hungarian adults (*n* = 431) during coronavirus lockdown compared to previous Hungarian scores and other countries’ residents’ scores during the COVID-19 pandemic.

The mean of participants’ perceived stress scores was 19.34 ± 7.97. The Hungarian adults were classified into three groups according to terciles (15, 23) of their perceived stress scores: low stress level (*n* = 130; 30.2%), medium stress level (*n* = 139; 32.3%), and high stress level (*n* = 162, 37.6%) groups. We compared this value with previous Hungarian and several other countries’ stress scores during the COVID-19 pandemic, and thosescores are illustrated in Figure 1.

The points of Hungarian adults were significantly higher than certain studies’ measured stress scores (19.34 ± 7.97): Hungarian adult participants of a stress management program in 2006 (M = 17.5; *p* < 0.01, t = 4.803); Hungarian healthy adults of the European Society for Dermatology and Psychiatry (ESDaP) Project 2 in 2019 (M = 17.76; *p* < 0.01, t = 4.126); Chinese adults in COVID-19 lockdown in February 2020 (M = 15.82; *p* < 0.01, t = 9.181); U.S.adults during the COVID-19 pandemic in June 2020 (M = 18.27; *p* < 0.01, t = 2.797); Colombian adults in COVID-19 lockdown in March 2020 (M = 16.5; *p* < 0.01, t = 7.409). Canadian adults during the COVID-19 pandemic in March–May 2020 showed significantly higher stress scores (M = 20.81; *p* < 0.01, t = −3.821) than the Hungarian sample of our study. Polish adults in COVID-19 lockdown in April 2020 (M = 19.22) stress scores’ did not differ significantly from Hungarian adults’ stress levels.

**Figure 2:**
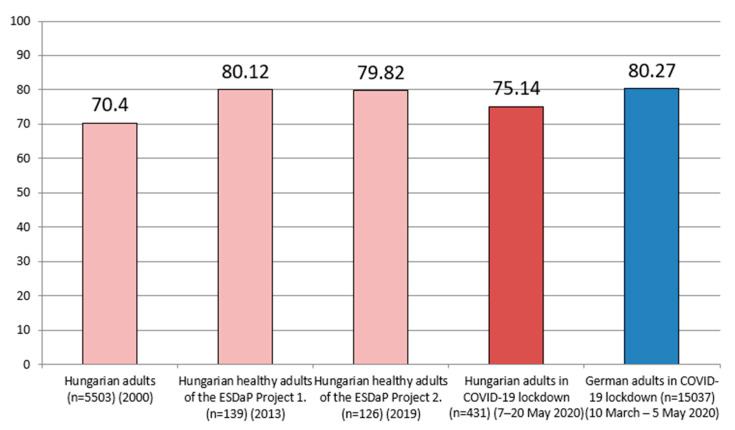
Health status results (measured with the EQ-VAS) of Hungarian adults without data with significant outliers (*n* = 418) during coronavirus lockdown compared to previous Hungarian scores and German residents’ scores during coronavirus lockdown.

The mean of participants’ health status scores was 73.05 ± 21.71. We compared this value with previous Hungarian and German adults’ health status scores in COVID-19 lockdown, which scores are illustrated in Figure 2. The third assumption of the one-sample *t*-test, that there should be no significant outliers, was violated for health status scores of the participants. Therefore, we eliminated the significant outliers of this variable (data of 13 participants) to be able to meet the third assumption of the test for this variable. Because of this, the mean of participants’ health status scores changed to 75.14 ± 18.48 for comparison of other studies’ scores.

The points of Hungarian adults were significantly lower than certain studies’ measured health status scores (75.14 ± 18.48): Hungarian healthy adults of the ESDaP Project 1 in 2013 (M = 80.12; *p* < 0.01, t = −5.514), Hungarian healthy adults of the ESDaP Project 2 in 2019 (M = 79.82; *p* < 0.01, t = −5.182), German adults in COVID-19 lockdown in March–May 2020 (M = 80.27; *p* < 0.01, t = −5.679). Hungarian adult participants of a National Health Survey in 2000 showed significantly lower health status scores (M = 70.4; *p* < 0.05, t = 5.240) than the Hungarian sample of our study.

**Figure 3:**
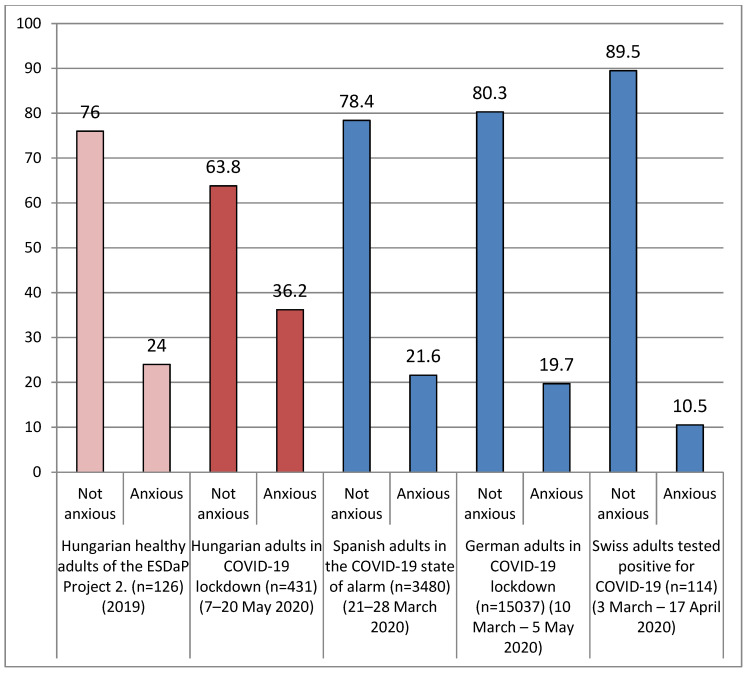
Frequencies of anxious versus not anxious cases (measured with the General Anxiety Disorder Assessment (GAD)-2 scale) at Hungarian adults (*n* = 431) during coronavirus lockdown compared to previous Hungarian scores and other countries’ residents’ scores during the COVID-19 pandemic.

The mean of participants’ anxiety scores was 2.3 ± 1.86. The Hungarian adults were classified into two groups: anxious (*n* = 156; 36.2%) and not anxious (*n* = 275; 63.8%) groups. We compared the frequencies of anxious and not anxious participants with a previous Hungarian score, Spanish adults’ in the COVID-19 state of alarm, German adults’ in COVID-19 lockdown, and Swiss adults that tested positive for COVID-19 anxiety scores, and these distributions are illustrated in Figure 3.

The points of Hungarian adults were significantly higher than certain studies’ measured anxiety scores (2.3 ± 1.86): Hungarian healthy adults of the ESDaP Project 2 in 2019 (M = 1.82; *p* < 0.01, t = 5.366), Spanish adults in the COVID-19 state of alarm in March 2020 (M = 1.79; *p* < 0.01, t = 5.701), German adults in COVID-19 lockdown in March–May 2020 (M = 1.52; *p* < 0.01, t = 8.709), and Swiss adults that tested positive for COVID-19 in March–April 2020 (M = 0.9; *p* < 0.01, t = 15.617).

**Figure 4:**
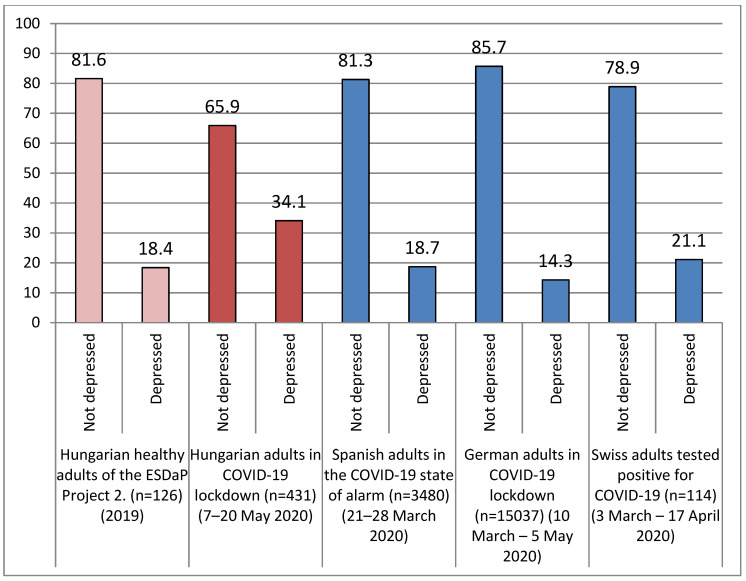
Frequencies of depressed versus not depressed cases (measured with the Patient Health Questionnaire (PHQ)-2 scale) at Hungarian adults (*n* = 431) during coronavirus lockdown compared to previous Hungarian scores and other countries’ residents’ scores during the COVID-19 pandemic.

The mean of participants’ depression scores was 2.19 ± 1.85. The Hungarian adults were classified into two groups: depressed (*n* = 147; 34.1%) and not depressed (*n* =284; 65.9%) groups. We compared the frequencies of depressed and not depressed participants with a previous Hungarian score, Spanish adults’ in the COVID-19 state of alarm, German adults’ in COVID-19 lockdown and Swiss adults that tested positive for COVID-19 depression scores, and those distributions are illustrated in Figure 4.

The points of Hungarian adults were significantly higher than certain studies’ measured depression scores (2.19 ± 1.85): Hungarian healthy adults of the ESDaP Project 2 in 2019 (M = 1.39; *p* < 0.01, t = 8.984), Spanish adults in the COVID-19 state of alarm in March 2020 (M = 1.6; *p* < 0.01, t = 6.633), German adults in COVID-19 lockdown in March–May 2020 (M = 1.14; *p* < 0.01, t = 11.783), and Swiss adults that tested positive for COVID-19 in March–April 2020 (M = 1.4; *p* < 0.01, t = 8.872).

Coping scores are illustrated in Table 3. The average percentage of scores for the subscales were: problem analysis, 60%; goal-oriented behavior, 48%; seeking emotional balance, 47%; withdrawal, 46%; asking for help, 44%; adaptation, 41%; and emotion-based behavior, 26%. The average percentage of scores for the two main scales of the instrument were: problem-focused coping, 53%; emotion-focused coping, 39%.

The mean of participants’ neurotic complaints score was 8.68 ± 4.65. 39% (*n* = 168) of the Hungarian adults had a neurotic personality trait, while 61% (*n* = 263) of them did not have the mentioned trait.

We summarized significant correlations between psychological factors’ scores of the participants in Table 4.

We would like to highlight some of the examined correlations here: perceived stress showed significant positive correlations with level of anxiety, level of depression, number of neurotic complaints, and emotion-focused coping, and it showed significant negative correlations with health status and problem-focused coping. Health status significantly positively correlated with problem-focused coping, and it significantly negatively correlated with perceived stress, level of anxiety, level of depression, number of neurotic complaints, and emotion-focused coping.

## 4. Discussion

In our study, we examined the psychological impact of the coronavirus pandemic on Hungarian adults by investigating their degree of perceived stress, levels of anxiety and depression, health status, number of neurotic complaints, forms of coping strategies, and subjective opinions regarding their mood and coping skills during the Hungarian lockdown in the Spring of 2020.

In our sample, 30.2% of participants belonged to the low stress level group, 32.3% to the medium stress group, and 37.6% to the high stress group. Stress scores of Hungarian adults were significantly higher than the following samples’ results: Hungarian adult participants of a stress management program (in 2006), Hungarian healthy adults of the ESDaP Project 2 (in 2019); Chinese adults in COVID-19 lockdown (in February 2020); U.S. adults during the COVID-19 pandemic (in June 2020); Colombian adults in COVID-19 lockdown (in March 2020). Our samples’ points of stress were significantly lower than the results of Canadian adults during the COVID-19 pandemic (in March-May 2020), and the Hungarian adults’ degree of perceived stress did not differ from Polish adults’ (in April 2020) in COVID-19 lockdown.

Health status scores (with an average score of 73.05 on a scale of 0 to 100) of our sample were significantly lower than Hungarian healthy adults of the ESDaP Project 1 (in 2013), Hungarian healthy adults of the ESDaP Project 2 (in 2019), and German adults in COVID-19 lockdown (in the Spring of 2020). It is worth noting that Hungarian adult participants of a National Health Survey in 2000 showed significantly lower health status scores than the sample of our study.

In our sample 36.2% of the participants were anxious, and 63.8% of them were not anxious based on their responses. Their anxiety scores were significantly higher than Hungarian healthy adults of the ESDaP Project 2 (in 2019), Spanish adults’ in the COVID-19 state of alarm (in March 2020), German adults’ in COVID-19 lockdown (in the Spring of 2020), and Swiss adults that tested positive for COVID-19 (in March–April 2020). According to the WHO Global Health Estimates [47] from 2015, 3.6% of the global population had anxiety disorders, and 3.9% of the Hungarian population. This data highlights that elevated levels of anxiety were measured in our sample.

In our study, 34.1% of the Hungarian adults were depressed, and 65.9% of them were not depressed based on their answers. Their depression scores were significantly higher than Hungarian healthy adults of the ESDaP Project 2 (in 2019), Spanish adults in the COVID-19 state of alarm (in March 2020), German adults in COVID-19 lockdown (in the Spring of 2020), and Swiss adults that tested positive for COVID-19 (in March–April 2020). The WHO Global Health Estimates [47] results showed that, in 2015, 4.4% of the global population had depression. Based on the publication of Eurostat-Health in the European Union [48], 4.9% of the Hungarian population reported that they had chronic depression in 2017. These data emphasizes that elevated levels of depression were measured in our sample. However, reporting a high degree of depression was not unprecedented in previous Hungarian epidemiological studies, for example, in the Hungarostudy 2002 31% of 45–64 year old Hungarians, and 41% of Hungarians over 65 years of age had symptoms suggestive of depression [49].

Regarding the coping results, the average percentage of scores for problem-focused coping was 53%, and 39% for emotion-focused coping. This value for the coping subscales were the following: problem analysis, 60%; goal-oriented behavior, 48%; seeking emotional balance, 47%; withdrawal, 46%; asking for help, 44%; adaptation, 41%; emotion-based behavior, 26%. Thirty-nine and nine-tenths percent of the Hungarian adults had a neurotic personality trait, and 61% of them did not have the specified trait. A mental health expert can help an individual in finding adaptive ways of coping with stress sources brought about by the quarantine situation. In 2014, the percentage of people in Hungary who reported having consulted a psychologist, psychotherapist, or psychiatrist in the 12 months prior to a study cited in the Eurostat-Health in the European Union [50] was 4% of the Hungarian population. It could be important to highlight the possibility of seeking mental help in Hungary during the pandemic.

We found significant correlations between all of the seven examined psychological variables of our study. Perceived stress significantly positively correlated with level of anxiety, level of depression, number of neurotic complaints, emotion-focused coping, and it significantly negatively correlated with health status and problem-focused coping. Health status showed significant positive correlations with problem-focused coping, perceived stress, level of anxiety, and level of depression, and it showed significant negative correlations with number of neurotic complaints and emotion-focused coping.

Resilience could be a factor that shaped psychological reactions of the examined Hungarian adults. Havnen et al. [51] found that Norvegian adults in March 2020 during the COVID-19 pandemic high on resilience reported lower levels of perceived stress, depression, and anxiety. Resilience can be described as a healthy adaptation despite adversity, and it involves of a group of factors which contribute to healthy coping and health promotion [51]. Havnen et al. [51] emphasized that people high on resilience were less affected by negative emotional reactions (such as increased worry and anxiety) due to the experience of stress during the coronavirus pandemic.

Our results highlight the importance of stress management in the psychological support of healthy adults in quarantine situation caused by the coronavirus pandemic. Reduction of anxiety and depression levels with the aid of psychologists and psychiatrists, forming coping strategies that affect quality of life may also help quarantined individuals. Positive changes in psychological factors in people may also lead to perceiving their health statuses to be better.

The present study has some limitations that are worth mentioning. There was an over-representation of female participants in our study; therefore, the possibility of volunteer bias needs to be considered, our results may not be representative of the Hungarian general adult population. Our project employed a cross-sectional design; hence, we cannot deduce causality between the examined variables. We intended to compare certain variables’ results of our study with several publications’ main outcomes where the authors used the same measuring instruments as we did, but we did not have the datasets of the cited articles, only means of the variables that we wished to compare with our results. Because of this, we could not use independent sample *t*-tests. Nonetheless, one-sample *t*-test was deemed suitable to compare a sample mean to a specific value. Three out of four assumptions were met to be eligible for analyzation with a one-sample *t*-test, although the one-sample *t*-test is robust to violations of normality to a certain degree. Based on the available data we decided to use one-sample *t*-tests, but the implications of these comparisons should be carefully considered. We would also like to mention strengths of our study. Cronbach’s alpha values of the used instruments mostly indicated a high level of internal consistency. We found significant correlations among all of the measured psychological variables of our project. These results can provide a strong foundation for tailoring psychological interventions for Hungarian people who are greatly affected psychologically from the coronavirus pandemic.

## 5. Conclusions

Our results highlight that Hungarian adults might have had elevated levels of stress, anxiety, and depression during the time of the nationwide lockdown in the Spring of 2020. Participants of our study tended to use problem-focused coping strategies more frequently than emotion-focused ones. Health status showed significant positive correlations with problem-focused coping, perceived stress, level of anxiety, level of depression, and it showed significant negative correlations with number of neurotic complaints and emotion-focused coping. We found significant correlations between all of the seven examined psychological variables of our study. Our results indicate the importance of stress management in the psychological support of healthy adults in quarantine situation caused by the coronavirus pandemic.

## Figures and Tables

**Table 1:** Details of studies which used the same measuring instruments as we did for comparison of results.

Author and Year	Time of Assessment	Location	The Assessment Tools (Relevant to Present Study)	Participants (Relevant to Present Study)	Age	Female	The Main Outcomes (Relevant to Present Study)
Bäuerle et al., (2020) [15]	10 March–5 May 2020	Germany	EQ-VAS; PHQ-2; GAD-2	adults residing in Germany in COVID-19 lockdown (*n* = 15037)	NA	70.07%	EQ-VAS = 80.27; PHQ-2: prevalence of 14.3% for depression; GAD-2: prevalence of 19.7% for anxiety
Chodkiewicz et al., (2020) [34]	10–20 April 2020	Poland	PSS-10	adult population in COVID-19 lockdown (*n* = 443)	31.9 ± 11.31	78.6%	PSS-10 = 19.22
González-Sanguino et al., (2020) [35]	21–28 March 2020	Spain	PHQ-2; GAD-2	general adult population in the COVID-19 state of alarm (*n* = 3480)	37.92	75%	PHQ-2: prevalence of 18.7% for depression; GAD-2: prevalence of 21.6% for anxiety
Nwachukwu et al., (2020) [36]	23 March–3 May 2020	Canada	PSS-10	adult population during the COVID-19 pandemic (*n* = 8267)	42.09 ± 3.44	87.1%	PSS-10 = 20.81 ± 6.83
Pedrozo-Pupo et al., (2020) [37]	21–23 March 2020	Colombia	PSS-10-C	adult population in COVID-19 lockdown (*n* = 406)	43.9 ± 12.4	61.8%	PSS-10-C = 16.5 ± 7.3
Shen et al., (2020) [38]	1–27 June 2020	USA	PSS-10	adults residing in the US during the COVID-19 pandemic (*n* = 800)	NA	83%	PSS-10= 18.27 ± 7.34
Speth et al., (2020) [39]	3 March–17 April 2020	Switzerland (Aarau, Kantonsspital Aarau)	PHQ-2; GAD-2	adults tested positive for COVID-19 (*n* = 114)	44.6 ± 16.1	54.4%	PHQ-2: prevalence of 21.1% for depression; GAD-2: prevalence of 10.5% for anxiety
Stauder & Konkoly-Thege (2006) [40]	2006	Hungary (Budapest)	PSS-10	adult participants of a behavioral-based stress management program (*n* = 69)	NA	NA	PSS-10 = 17.5 ± 6.0
Szabó et al., (2016) [41,42]	2011–2013	Hungary (Szeged)	EQ-VAS	hospital employees at the research institution (*n* = 139)	39.53 ± 2.01	75%	EQ-VAS = 80.12 ± 17.76
Szabó et al., (2019) [43,44]	2017–2019	Hungary (Szeged)	EQ-VAS; PHQ-2; GAD-2; PSS-10	hospital staff members of the research institution (*n* = 126)	38.94 ± 2.31	82%	EQ-VAS = 79.82 ± 16.13; PSS-10 = 17.76 ± 5.39; PHQ-2: prevalence of 18.4% for depression; GAD-2: prevalence of 24% for anxiety
Szende & Németh (2000) [45]	2000	Hungary	EQ-VAS	survey was part of the National Health Survey conducted on a representative sample of members of the general population (*n* = 5503)	NA	NA	EQ-VAS = 70.4
Zhao et al., (2020) [46]	18–25 February 2020	China	PSS-10	non-diseased members of the general public in COVID-19 lockdown (*n* = 1630)	29.17 ± 10.58	NA	PSS-10 = 15.82 ± 5.56

**Table 2:** Participants’ attributes (*n* = 431). Optionally answerable items of the questionnaire were marked with a * symbol.

		Hungarian Adult Participants *n* (%)
Gender	Male	44 (10%)
	Female	387 (90%)
Age	Mean ± SD	47.53 ± 11.66
	Range	18–73 years
Marital status *	Single	61 (14%)
	Married/In a relationship	291 (68%)
	Divorced	54 (13%)
	Widowed	18 (4%)
	Other	1 (0.2%)
Level of education *	Primary school (1–8 grades)	5 (1.1%)
	Vocational school (9–12 grades)	34 (8%)
	High school (9–12 grades)	53 (12%)
	Secondary school (9–12 grades)	97 (23%)
	University, college	226 (52%)
	PhD, DLA	6 (1.4%)
	Other	5 (1.1%)
Employment status	Currently employed	298 (69%)
	Student	5 (1%)
	On sick leave	6 (1%)
	Retired	72 (17%)
	Unemployed	50 (12%)

**Table 3:** Frequencies of coping strategies (measured with the Shortened (Hungarian) version of the Ways of Coping Questionnaire (WOC)) of Hungarian adults (*n* = 431) during coronavirus lockdown.

	Mean	SD	Range of Subscale	Average Percentage (Mean/Total Score of Subscale × 100)
Problem analysis	5.43	1.78	0–9	60
Goal-oriented behavior	5.78	2.78	0–12	48
Emotion-based behavior	3.13	2.35	0–12	26
Adaptation	4.92	1.82	0–12	41
Asking for help	2.64	1.42	0–6	44
Seeking emotional balance	2.8	1.35	0–6	47
Withdrawal	4.13	1.89	0–9	46
Problem-focused coping	11.21	3.90	0–21	53
Emotion-focused coping	17.62	5.24	0–45	39

**Table 4:** Significant correlations between psychological factors’ scores of Hungarian adults (*n* = 431) during coronavirus lockdown (Spearman rank-order correlation coefficients ** *p*<0.01).

	Perceived Stress	Level of Anxiety	Level of Depression	Health Status	Number of Complaints	Problem-Focused Coping	Emotion-Focused Coping
Perceived stress	-	0.821 **	0.778 **	−0.448 **	0.626 **	−0.330 **	0.400 **
Level of anxiety	0.821 **	-	0.753 **	−0.399 **	0.544 **	−0.240 **	0.357 **
Level of depression	0.778 **	0.753 **	-	−0.416 **	0.501 **	−0.243 **	0.329 **
Health status	−0.448 **	−0.399 **	−0.416 **	-	−0.496 **	0.210 **	−0.195 **
Number of complaints	0.626 **	0.544 **	0.501 **	−0.496 **	-	−0.304 **	0.372 **
Problem-focused coping	−0.330 **	−0.240 **	−0.243 **	0.210 **	−0.304 **	-	0.161 **
Emotion-focused coping	0.400 **	0.357 **	0.329 **	−0.195 **	0.372 **	0.161 **	-
